# Novel antibodies detect additional α-synuclein pathology in synucleinopathies: potential development for immunotherapy

**DOI:** 10.1186/s13195-020-00727-x

**Published:** 2020-11-30

**Authors:** Jacqui T. Nimmo, Ajay Verma, Jean-Cosme Dodart, Chang Yi Wang, Jimmy Savistchenko, Ronald Melki, Roxana O. Carare, James A. R. Nicoll

**Affiliations:** 1 Clinical Neurosciences. Clinical & Experimental Sciences, Faculty of Medicine, University of Southampton, Southampton, UK; 2 United Neuroscience, Dublin, Republic of Ireland; 3 Institute Francois Jacob (MIRCen), CEA and Laboratory of Neurodegenerative Diseases, CNRS, Paris, France

**Keywords:** Immunotherapy, Synucleinopathies, Pathology, Parkinson’s disease, Dementia with Lewy bodies, Multiple system atrophy

## Abstract

**Background:**

Alpha-synuclein (α-Syn) aggregation is the primary characteristic of synucleinopathies including Parkinson’s disease (PD), dementia with Lewy bodies (DLB) and multiple system atrophy (MSA). Immunotherapy targeting α-Syn has shown promising results in animal models of the disease. This study investigates the target specificity of three different active vaccines for pathological α-Syn aggregates found in human brain tissue from synucleinopathies.

**Methods:**

Guinea pigs were immunised with 3 vaccines developed by United Neuroscience, and IgG fractions purified from the resulting immune sera (IGG-1, IGG-2 or IGG-3) were used to perform immunohistochemical staining of human cases of PD, DLB and MSA. The resulting immunoreactivity was compared to a commercially available α-Syn antibody from Novacastra (NOV) commonly used for diagnostic purposes. Images were captured from the substantia nigra (SN), temporal lobe, internal capsule, insular cortex and putamen and quantified for the percentage area with α-Syn immunoreactivity. Lewy bodies (LB) and Lewy neurites (LN) were further analysed in PD and DLB cases.

**Results:**

Vaccine-generated antibodies detected more α-Syn pathology compared to NOV. The levels of α-Syn immunoreactivity varied between brain region and disease type with IGG-3 recognising the highest levels of α-Syn in most cases and in all brain regions that are affected early in disease progression. IGG-3 had a high recognition for glial inclusions found in MSA which are known to have a more compact conformation. Slot blot analysis confirmed the specificity of IGG-3 for native oligomers and fibrillar α-Syn. Higher levels of α-Syn were recognised by IGG-2 in cortical regions, and by IGG-3 in SN of PD and DLB cases. This was due to increased immunolabelling of LNs in these brain regions suggesting that IGG-2 and IGG-3 recognised additional α-Syn pathology compared to IGG-1 and NOV. Whether the unique binding properties of the antibodies produced in guinea pigs will translate in the clinic remains to be addressed, which is the main limitation of this study.

**Conclusions:**

These vaccines induce antibodies that bind α-Syn oligomers and aggregates in the human brain and specifically support the choice of the vaccine generating IGG-3 (i.e. UB-312) as a candidate for clinical trials for synucleinopathies.

## Introduction

There is currently no treatment to halt the progression of the group of neurodegenerative diseases comprising the synucleinopathies. Immunotherapies have advanced significantly in recent years, demonstrating promising results in rodent models of neurodegeneration as well as in some clinical trials [1]. The majority of immunotherapy research has been focused on Alzheimer’s disease (AD) with less attention given to other neurodegenerative diseases. Despite this, there is growing progress towards developing vaccines against alpha synuclein (α-Syn) with at least three anti-α-Syn vaccines in early phase clinical trials demonstrating good safety profiles in patients with Parkinson’s disease (PD) [1].

The most common synucleinopathies include Parkinson’s disease (PD), Dementia with Lewy bodies (DLB) and multiple system atrophy (MSA) in which α-Syn plays a central role in the progression of neurodegeneration [2, 3]. In each disease, the anatomical distribution and progression of α-Syn aggregation correlates with clinical symptoms [4]. According to Braak staging, α-Syn aggregation is first observed in the medulla and olfactory structures in PD and DLB which then progresses through the brainstem, with characteristic loss of pigmented dopaminergic neurons in the substantia nigra (SN), to the limbic, temporal mesocortex and neocortical regions [5, 6]. Abnormal aggregation of α-Syn occurs progressively within neuronal perikarya and neurites to form α-Syn rich Lewy bodies (LB) and Lewy neurites (LN) respectively [7]. Autonomic dysfunction is also a typical characteristic in PD and DLB, and this is associated with α-Syn pathology found in LB inclusions in the peripheral nervous system, in particular the enteric nervous system [8]. Unlike PD and DLB, the accumulation of α-Syn in MSA patients occurs within glial cells as glial cytoplasmic inclusions (GCI) [9]. α-Syn accumulation in MSA is more widespread than PD and DLB leading to a faster and more aggressive disease progression [9].

The aggregation of α-Syn is a multistep process involving oligomerisation of monomeric α-Syn into insoluble fibrils and finally larger aggregates [10]. α-Syn oligomers or fibrils can act as ‘seeds’ for the propagation of α-Syn aggregation in surrounding cells [11, 12]. The conformation of α-Syn oligomers and larger aggregates within α-Syn-rich deposits [13] varies depending on the intracellular milieu and brain region affected, such that oligodendrocyte α-Syn has been found to propagate much faster than neuronal α-Syn (hence the aggressive nature of MSA) and has a compact structure that is resistance to degradation by proteinase K [12]. Similarly cortical Lewy Bodies (LB) are known to adopt a smaller structure than LBs found in the SN and lack the distinct halo that characterises nigral LBs, suggesting that these may have different conformations of α-Syn [14].

The identification of natural antibodies against α-Syn in PD patients [15–17] suggests that the immune system could be involved in the clearance of pathogenic α-Syn. Similarly, memory B cells isolated from PD patients produced a repertoire of anti-α-Syn antibodies [15]. Those antibodies with highest affinities were all directed to the C-terminus of α-Syn and were able to prevent the propagation of α-Syn aggregation in vitro [15]. This mechanism of antibody-aided clearance is likely to be mainly by targeting extracellular forms of α-Syn, which are more accessible than intracellular aggregates [18]. The neuroprotective antibodies were found to decrease with ageing, perhaps increasing the susceptibility of aged individuals to neurodegeneration [19, 20]. Immunotherapy is therefore aimed at the enhancement of α-Syn clearance in the brain by mimicking the natural protective immunity, and restoring these antibody levels in aged individuals [21]. Preclinical studies using immunotherapy in mouse models of synucleinopathies have demonstrated the potential for anti-α-Syn vaccines to prevent propagation of α-Syn pathology, enhance clearance of α-Syn aggregates, reduce neuronal loss and ameliorate behavioural deficits [22–25].

Retrospective review of the many AD immunotherapy clinical trials, particularly the AN1792 Elan trial [26, 27], has provided insight into vaccine design to achieve good safety profiles by preventing the immune response to involve activation and infiltration of pro-inflammatory Th1-cells [27]. A major challenge to developing vaccines against neurodegenerative diseases is that the targeted antigen is a self-peptide in which only structural differences distinguish the pathogenic from the normal form of the peptide [21]. Overcoming this ‘self’ barrier, while avoiding overactivation of pro-inflammatory T cells and obtaining high antibody titres in an ageing and immune compromised population, is challenging. The study described here relates to the development of novel vaccines against α-Syn with improved functional antigenicity and immunogenicity (United Neuroscience) [28]. This vaccine is composed of a fully synthetic peptide, in which intrinsic self-Th cell epitopes are replaced by foreign promiscuous UBITh Th-cell epitopes that are covalently linked to the functional antigenic peptides (such as α-Syn) by peptide synthesis. Use of foreign T cell epitopes increases the immunogenicity of the functional antigenic peptides and reduces the need for use of strong adjuvants to elicit an immune response. UBITh therefore enhances the B cell response to specifically produce site-directed antibodies against α-Syn. Thus, the UBITh platform specifically modulates components of the immune system in a way not done before. Pre-clinical in vivo studies showed no detection of microglial or astrocyte activation and no infiltration of CD3+, CD4+, or CD8+ T cells. UBITh-based vaccines have been developed against Aβ (UB-311) and have completed phase II clinical trials in mild to moderate AD patients (NCT02551809) [28]. These clinical trials indicated good safety and tolerability and a very high responder rate, which is not seen in most other vaccines. In general, active immunisation protocols for protein aggregation disorders have substantial advantages over passive immunisation because they do not require manufacturing of large amounts of antibody, do not need to be given as regular infusions and have longer duration of action [29].

This study aims to explore the recognition of pathological forms of α-Syn in human cases of PD, DLB and MSA when different α-Syn epitopes are employed in a UBITh-based vaccine [28]. Such information will further support the rationale in the development of UBITh-based α-Syn vaccines, already completed a phase I clinical trial in healthy volunteers and is on the way to be tested in patients with Parkinson’s disease (UB-312, NCT04075318).

## Materials and methods

### Vaccines and production of anti-α-Syn antibodies in guinea pigs

Over 60 different α-Syn peptide antigens (B-cell epitopes) conjugated to UBITh® peptides were screened for their immunogenicity in guinea pigs [28]. From these studies, three immunogens/vaccines (VX-01, VX-02 and VX-03) directed against overlapping amino acid (AA) sequences at the C-terminal region of α-Syn, with antigens of 21 AA, 24 AA and 12 AA respectively, were selected for investigation of their potential to induce antibodies with specificity for pathological forms of α-Syn [28, 30]. Each vaccine targets a different epitopic region of the α-Syn protein and shows no homology to other synucleins [28].

Duncan-Hartley Guinea pigs (300–350 g) were immunised (3 intramuscular injections, 3 weeks apart) with the UBITh-based vaccines VX-01, VX-02 and VX-03. Sera were collected 9 weeks after the prime injection and pooled within each group (*n* = 3 animals per group). The total IgG fraction was isolated from each of the pooled sera using a Protein A IgG Purification Kit (ThermoFisher, #44667) and were each normalised to 1 mg/mL after using a Bicinchoninic Acid (BCA) Protein Assay. The purified IgG fraction derived from vaccinations with VX-01, VX-02 and VX-03 are labelled IGG-1 (PD062220-09-I-2-Syn), IGG-2 (PD062205-09-I-2-Syn) and IGG-3 (PD100806-09-I-2-Syn) respectively. In certain occasions, sera (100 μg/mL) were used instead of total IgG fractions.

### Human cases of synucleinopathies

Tissue specimens were obtained from the Parkinson’s UK Brain Bank at Imperial College, London (ethical approval reference number 18/WA/0283), from cases with multiple system atrophy (MSA), dementia with Lewy bodies (DLB) and Parkinson’s disease (PD) (*n* = 12 in total, Table 1). Cases without significant neuropathology were used as controls (*n* = 3). Sections of formalin-fixed and paraffin-embedded brain tissue 10 μm in thickness were used. Brain regions analysed included the basal ganglia (putamen, internal capsule and adjacent insular cortex), midbrain (substantia nigra), and temporal lobe (neocortex and white matter). These brain regions are known to be affected by α-Syn aggregation in varying degrees and at various stages of the disease progression in each disease type. Generally, the basal ganglia and midbrain are affected at mid-stages in DLB, PD and MSA and hence also have the highest aggregate burden. The temporal cortex is affected at later stages of disease.

**Table 1:** List of human cases of synucleinopathies

Case ID	Age at death (years)	Sex	Age at onset (years)	Duration (years)	PMI (hours)	Neuropathological diagnosis *
MSA						
PD363	61	F	48	13	21	Multiple system atrophy
PD505	90	M	87	3	22	Multiple system atrophy
PD300	75	M	68	7	26	Multiple system atrophy (striato-nigral dominant)
DLB						
PD294	78	M	72	6	24	Lewy body disease (alpha-synucleinopathy), early neocortical type
PD385	80	M	75	5	19	Lewy body disease (alpha-synucleinopathy), neocortical type
PD330	65	F	71	5	?	Lewy body disease (alpha-synucleinopathy), neocortical type
PD						
PD433	74	F	55	19	29	Lewy body disease (alpha-synucleinopathy), neocortical type
PD458	73	M	54	19	10	Lewy body disease (alpha-synucleinopathy), neocortical predominant, severe
PD413	72	F	51	21	14	Lewy body disease (alpha-synucleinopathy), neocortical type
Control						
PDC29	82	M	–	–	48	–
PDC30	77	M	–	–	47	–
PDC92	79	M	–	–	25	–

*ACTA NEUROPATHOL 2009, 117:635–652

### Immunohistochemistry

IGG-1, IGG-2 or IGG-3 antibodies were used for IHC in order to compare the levels of α-Syn pathology detected. The antibodies were also compared to a commercially available antibody NOV (NCL-L-ASYN 1:100, Novacastra) commonly used for diagnostic purposes.

Paraffin-embedded tissue sections were dewaxed in a 60 °C oven for 15–20 min and then in Xylene for 10 min. The tissue was rehydrated in 4 dilutions of industrial methylated spirit (IMS, M/4450/17 Fisher Scientific) from 100 to 50% for 5 min each. The tissue was incubated for 3 min in 100% formic acid (ThermoFisher). Endogenous peroxidase activity was quenched with 3% H_2_O_2_ (H1009-500ML, Sigma Aldrich) for 10 min. Heat-induced antigen retrieval was performed by heating the tissue in citrate buffer (15 mM Tris sodium citrate [101578237, Sigma Aldrich], 0.1% tween, pH 6 [P1379, Sigma Aldrich]) using a Panasonic 800 W microwave at medium heat for 25 min. Non-specific binding sites were blocked with 15% normal goat serum (Fisher Scientific) for at least 30 min. The tissue was then incubated overnight at 4 °C with IGG-1, IGG-2, IGG-3 or the monoclonal antibody NOV in 1:100 dilution in 0.01 M PBS, 0.1% triton X [1001466726, ThermoFisher]. The following day, the tissue was incubated for 1 h in biotinylated goat anti-mouse or goat anti-guinea pig secondary antibodies at room temperature (RT). Tissue was incubated in Avidin biotin complex (ABC) for 1 h at RT (PK-6100 Vectastain ABC kit). Development of the chromogen was performed using VIP peroxidase (SK-4605 ImmPACT-VIP peroxidase kit) as detailed in the manufacturer’s instructions. Prior to mounting in Distyrene Plasticizer Xylene (DPX, 12658646 Fisher Scientific) mountant, the tissue was dehydrated for 2 min each in IMS 50%, 70%, 95%, 100%, and Xylene.

### Image analysis and statistics

Slides were scanned for analysis at × 20 objective using either an Olympus VS110 high throughput Virtual Microscopy System or Olympus dot Slide Virtual Microscopy System. Thirty images (each 0.25 mm^2^) were captured from the scanned image using *Olympus* VS *software*. The percentage area of α-Syn detected with each antibody was calculated using FIJI software. This gives thirty repeated measures of percentage area for each brain region. To enable comparison of results between antibodies, the images were taken from the same anatomical regions in each case.

To compare levels of LBs and LNs detected by each preparation of antibodies, the percentage area of LBs was calculated based on their size (1.03 μm^2^-Infinity) and circularity (0.6–1.0, where 1.0 is a perfect circle) in the same thirty images used above the SN and temporal lobe grey matter of PD and DLB cases. The percentage area of LNs was calculated by subtracting the percentage area of LBs from the total percentage of α-Syn immunoreactivity. While the remainder of α-Syn staining consisted mainly of LNs, some diffuse neuronal and neuropil immunoreactivity also contributed to this proportion.

Statistical analysis was conducted using SPSS V25 software. A univariate analysis was conducted to compare antibody detection of α-Syn within each brain region. Post hoc analysis was conducted with Bonferroni corrections for multiple comparison analysis. Differences were considered as significant when *p* < 0.05. Numbers (*n*) refer to the number of cases used for each experiment.

### In vitro binding to α-Syn monomers, oligomers and different fibrillar polymorphs

We compared the affinity of IGG-3 antibodies and the commercial antibody Syn1 (clone 42, BD Bioscience) for distinct species of alpha-synuclein assemblies using a filter trap assay. The α-Syn assemblies we used were structurally distinct fibrillar polymorphs (fibrils, ribbons, fibrils 65, fibrils 91), a fibrillar form lacking the 30 C-terminal amino acid residues of α-Syn (fibrils 110), α-Syn oligomers (O550) dopamine stabilised (ODA) and glutaraldehyde stabilised (OGA) oligomers, all on fibrillar assembly pathway, as previously described [31, 32]. Monomeric α-Syn, purified as described previously [33], was used as a control. Increasing amounts of fibrillar, oligomeric or monomeric α-Syn in the range 20 pg to 200 ng were spotted on nitrocellulose filters (Protran 0.45 μm NC) using a slot blot filtration apparatus (GE-80-6095-58, GE Healthcare). The filters were next blocked with skimmed milk, incubated with the indicated antibody at the indicated dilution. After extensive washing, we revealed the primary antibody binding profile using a rabbit anti-Guinea pig IgG (H+L) secondary antibody-HRP conjugate (61-4620, ThermoFisher) and Syn1 using a goat-anti-mouse secondary antibody (GTX213111-01, Gentex). Controls with secondary antibodies only were also included in the study. Super Signal ECL (34096, Pierce) was used. The blots were imaged on a BioRad imager (Chemidoc MP imaging system/BioRad imagelab software).

## Results

### Vaccine-generated antibodies recognise pathological forms of human α-Syn

The specificity of vaccine-generated antibodies for pathological forms of α-Syn present in synucleinopathies was investigated by IHC on human brain tissue from cases with pathologically confirmed PD, DLB, MSA and age-matched controls. Purified IgG fractions (antibodies) IGG-1, IGG-2 and IGG-3 from the vaccinated animals were used to stain the brain tissue for α-Syn, and the resulting staining was compared to the commercial NOV antibody.

**Fig. 1:**
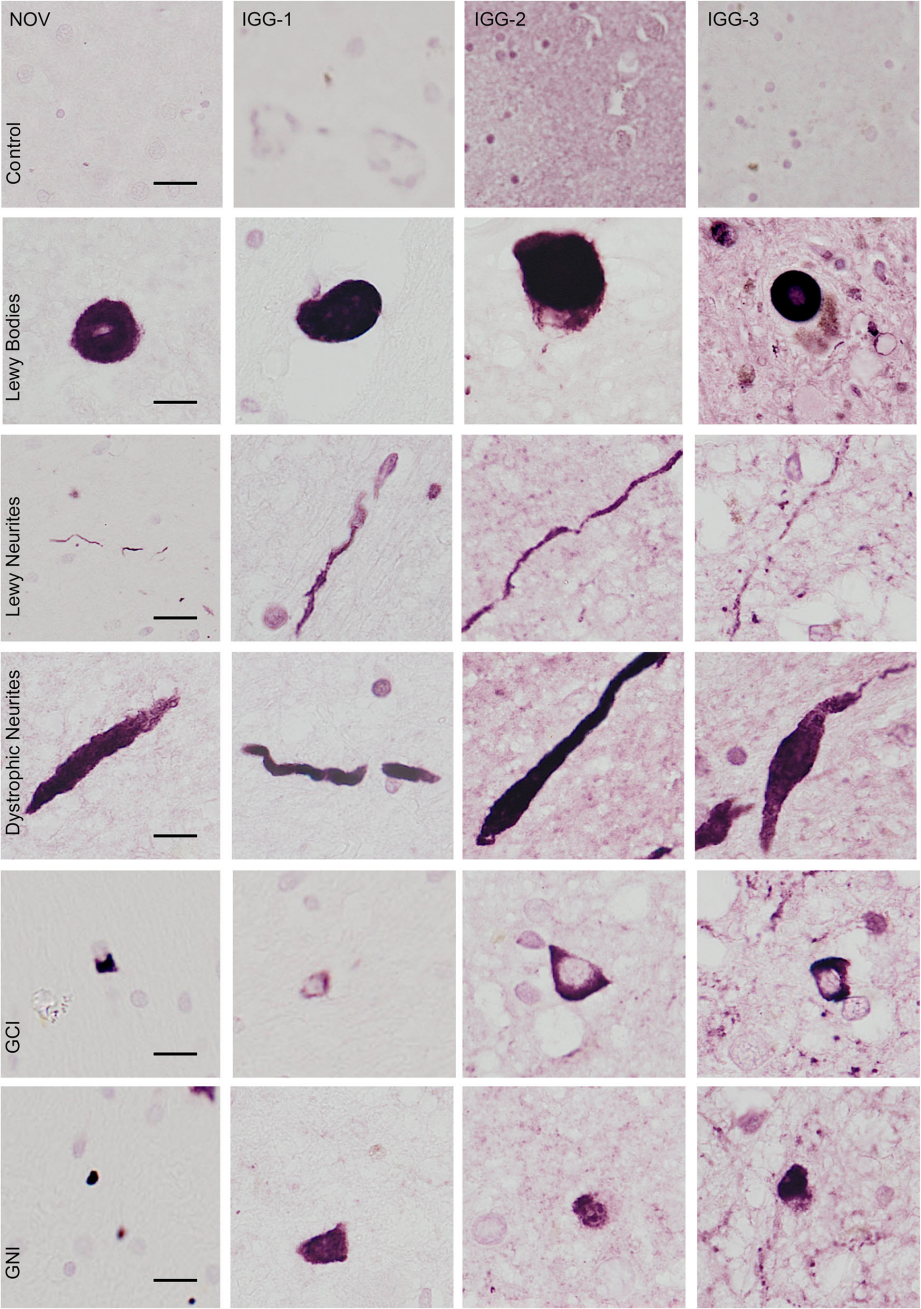
IHC analysis of characteristic α-Syn aggregates in synucleinopathies. Representative images of Lewy bodies, Lewy neurites and dystrophic neurites in LB diseases (PD and DLB), and glial cytoplasmic inclusions (GCI) and glial nuclear inclusion (GNI) in cases of MSA are shown for IGG-1, IGG-2, IGG-3 and NOV. Top panel shows lack of α-Syn immunoreactivity in control cases for each antibody. Scale bar = 10 μm

IGG-1, IGG-2 and IGG-3 detected characteristic aggregates found in PD, DLB and MSA including LBs, LNs, dystrophic neurites, GCI and glial nuclear inclusions (GNI) (Fig. 1). The overall morphologies of the aggregates were similar to NOV. None of the immune sera, antibodies or NOV antibody showed any immunoreactivity in the age-matched controls demonstrating their highly specific immunoreactivities.

### Comparison of vaccine-generated antibodies across diseases and brain regions

The relative immunoreactivities of IGG-1, IGG-2 and IGG-3 were compared to NOV in brain regions that are affected at different stages in disease progression of the different synucleinopathies. The brain regions selected included substantia nigra, putamen, internal capsule, insular cortex and temporal lobe (grey and white matter). Generally, the antibodies detected significantly higher levels of α-Syn aggregates than NOV.

### Parkinson’s disease

**Fig. 2:**
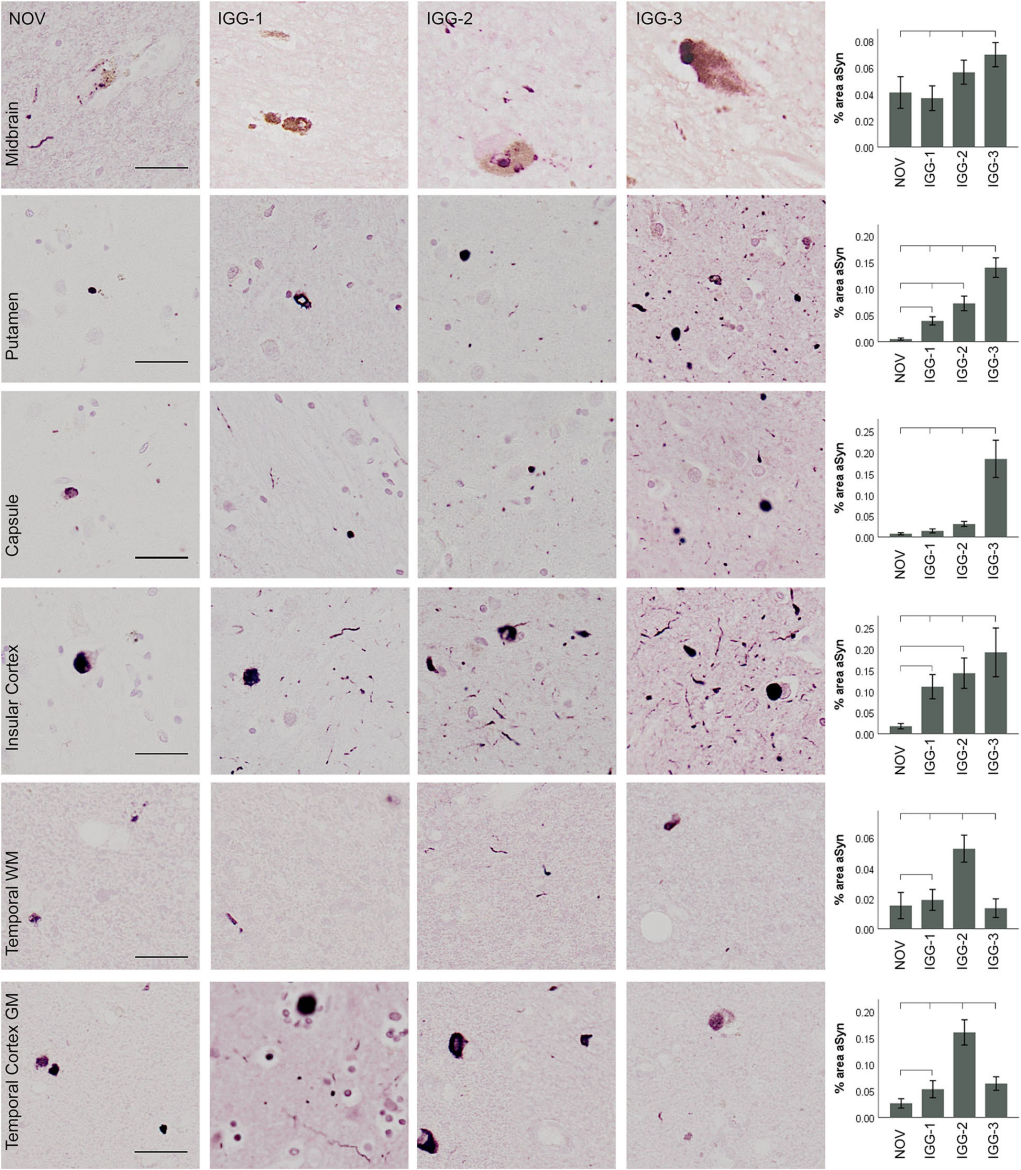
α-Syn immunoreactivity in Parkinson’s disease. Representative images and corresponding quantification of α-Syn immunoreactivity obtained using IGG-1, IGG-2, IGG-3 and NOV in different brain regions. α-Syn-positive inclusions were detected with both NOV and antibodies in each brain region. IGG-1, IGG-2 and IGG-3 detected significantly higher quantity of α-Syn inclusions than NOV in the putamen, insular cortex, and temporal cortex (*P* < 0.05). There was no difference between the % area of α-Syn detected by IGG-1 or IGG-2 and NOV in the substantia nigra and internal capsule. IGG-3 detected a significantly higher quantity than NOV in the substantia nigra, basal ganglia and cortical GM (*P* < 0.0001), and a significant increase was also seen compared to IGG-1 and IGG-2 in substantia nigra, and IGG-2 and in the basal ganglia (*P* < 0.0001). α-Syn detected by IGG-2 was significantly higher than IGG-3 in temporal cortex (*P* = 0.0001) and IGG-1 in temporal white matter (*P* < 0.0001). Error bars show mean ± 95% CI. Scale bar = 100 μm

In PD, α-Syn-positive inclusions were detected with NOV, IGG-1, IGG-2, and IGG-3 in each brain region (Fig. 2). IGG-1, IGG-2 and IGG-3 detected significantly more α-Syn inclusions than NOV in the putamen, insular cortex and temporal cortex (*P* < 0.05). There was no difference between the % area of α-Syn detected by IGG-1 or IGG-2 and NOV in the substantia nigra and internal capsule. IGG-3 detected a significantly higher quantity than NOV in the substantia nigra, basal ganglia and temporal cortex (*P* < 0.0001). The results for each of the antibodies are not consistent across brain regions. In the substantia nigra IGG-3 detected significantly higher levels of α-Syn than IGG-1 (*P* < 0.0001). In the putamen, internal capsule and insular cortex, IGG-3 also detected higher levels of α-Syn than IGG-1 and IGG-2 (*P* < 0.02). As expected, there was notably less α-Syn in the white matter of the temporal lobe compared to cortical grey matter and other brain regions. Despite this, in both grey and white matter, IGG-2 demonstrated highest sensitivity for α-Syn (*P* < 0.001). IGG-1 showed the lowest levels of α-Syn detection in all brain regions except temporal white matter.

### Dementia with Lewy bodies

In DLB, all antibodies were significantly more sensitive to α-Syn detection, when compared to NOV, in all brain regions, except for IGG-1 in the substantia nigra and IGG-3 in the temporal white matter. There was no consistent trend in the levels of α-Syn detected by each of the antibodies, which varied depending on the brain region. IGG-3 detected significantly higher levels of α-Syn than IGG-1 and IGG-2 in the substantia nigra and putamen (*P* < 0.0001). In the insular cortex, IGG-2 detected the highest levels of α-Syn and this was significantly greater than IGG-1. In the grey matter of the temporal lobe, IGG-2 immunoreactivity was significantly higher than that of IGG-3 and IGG-1. Similar to PD, there is much less α-Syn pathology in white matter regions. In contrast to the temporal cortex, IGG-1 detected significantly higher levels of α-Syn than IGG-3 and IGG-2 (*P* < 0.0001) in the temporal white matter.

### Multiple system atrophy

**Fig. 3:**
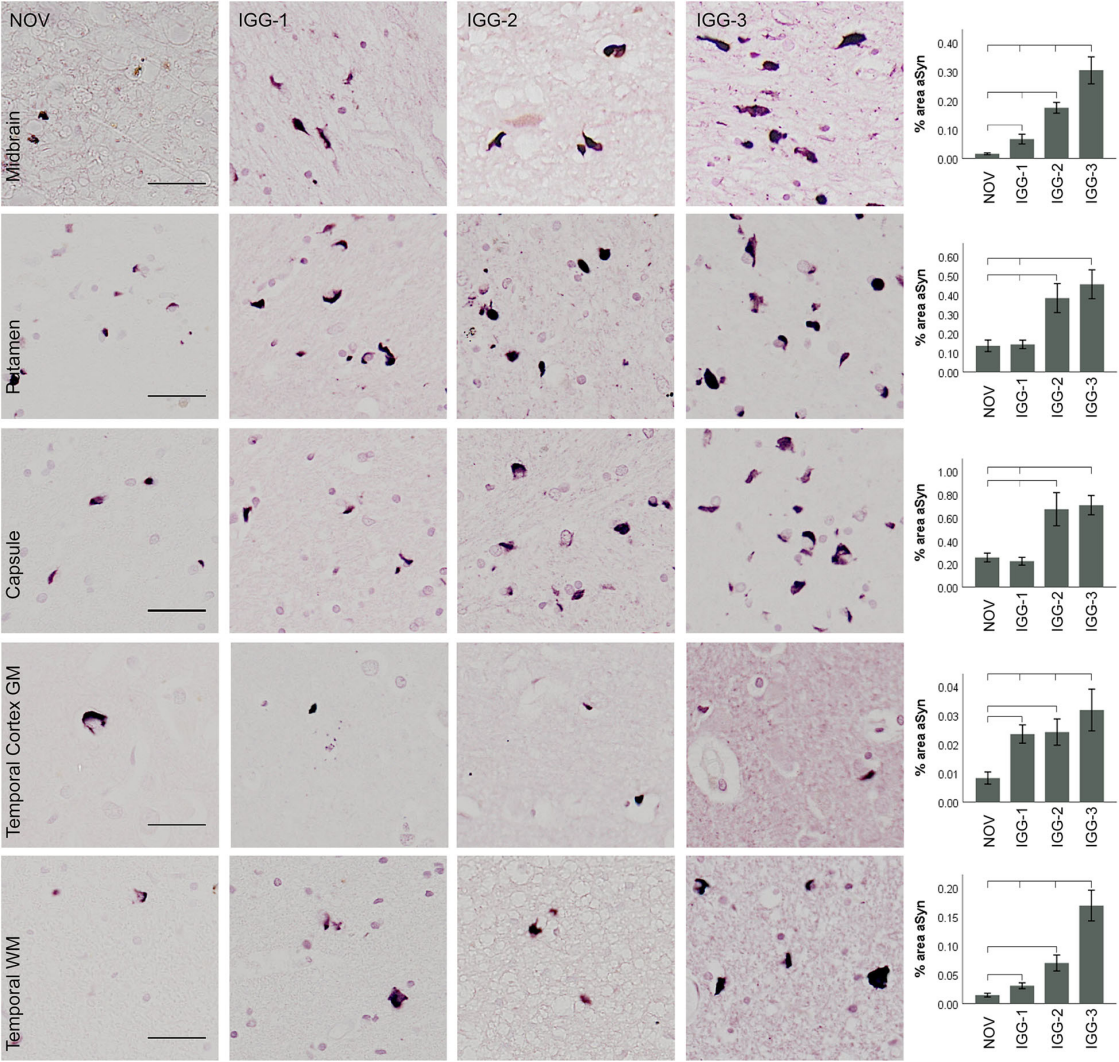
α-Syn immunoreactivity in multiple system atrophy. Representative images and corresponding quantification of α-Syn immunoreactivity obtained using IGG-1, IGG-2, IGG-3 and NOV. α-Syn-positive inclusions were detected with both NOV and all 3 antibodies in each brain region. IGG-1, IGG-2 and IGG-3 detected significantly a higher quantity of α-Syn inclusions than NOV in the substantia nigra and temporal lobe GM and WM (*P* < 0.05). IGG-2 and IGG-3 detected a significantly higher quantity than NOV and IGG-1 in all brain regions (*P* < 0.05). α-Syn detected by IGG-3 was significantly higher than IGG-2 in the substantia nigra and temporal lobe GM and WM (*P* > 0.0001). Error bars show mean ± 95% CI. Scale bar = 100 μm

IGG-1, IGG-2 and IGG-3 demonstrated significantly higher sensitivity for α-Syn aggregates than NOV in all brain regions (Fig. 3), with the exception of IGG-1 in the basal ganglia. In the substantia nigra, basal ganglia and temporal lobe, IGG-3 detected the highest percentage of α-Syn, with a 2–5-fold increased detection when compared to NOV. α-Syn immunoreactivity with IGG-3 was significantly different to IGG-1 in each brain region (*P* < 0.0001) as well as IGG-2 in the substantia nigra (*P* < 0.0001) and subcortical white matter (*P* < 0.05). In the white matter in particular, α-Syn immunoreactivity appeared on morphological grounds to be largely confined to glial cells, unlike in PD and DLB in which it was mainly present in neuronal processes.

### Vaccine-generated antibodies recognise a higher proportion of Lewy neurites compared to NOV

**Fig. 4:**
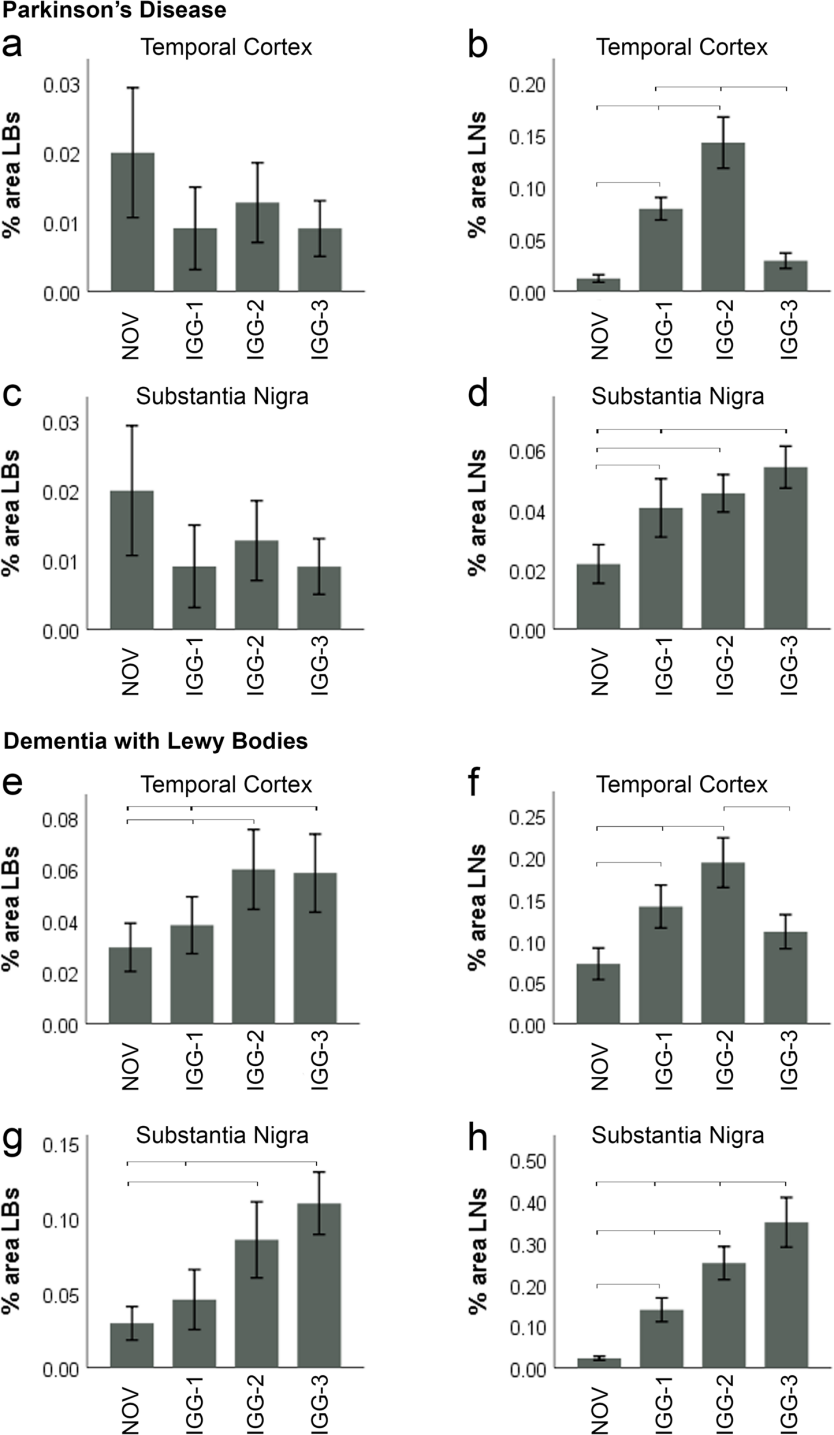
Quantification of Lewy bodies and Lewy neurites in PD and DLB. IGG-1, I GG-2, IGG-3. No difference in % area LBs in the temporal cortex and SN of PD cases was observed between each of the antibodies and NOV (**a**, **c**). **b** However, significantly more LNs were detected with IGG-2 and IGG-1 antibodies compared to IGG-3 and NOV (*P* < 0.0001); in the TC, there was no difference between IGG-3 and NOV. In addition, IGG-2 stained more LNs than IGG-1 in the temporal cortex of PD cases (*P* < 0.0001). **d** In the SN of PD cases, the % area LNs was greater for IGG-1 (*P* = 0.002), IGG-2 (*P* < 0.0001) and IGG-3 (*P* < 0.0001) compared to NOV. in addition IGG-3 was significantly greater than IGG-1 (*P* = 0.028) (**d**). **e** % area LBs detected by IGG-3 (*P* = 0.006) and IGG-2 (*P* = 0.001) was significantly greater than NOV. In addition, IGG-2 stained more LBs than IGG-1 in TC of DLB cases (*P* = 0.014). **f** Significantly more LNs were detected with IGG-2 compared to IGG-1, IGG-3 and NOV (*P* < 0.0001). In addition, IGG-1 stained more LNs than NOV in TC of DLB cases (*P* < 0.0001), but there was no difference between IGG-3 and NOV. **g** IGG-2 (*P* = 0.001) and IGG-3 (*P* < 0.0001) showed greater % area LBs in SN of DLB cases compared to NOV. IGG-3 also detected a higher level of LBs compared to IGG-1 (*P* = 0.002). **h** All antibodies showed significantly higher detection of LNs in SN compared to NOV in which the greatest difference occurred with IGG-3 (*P* < 0.0001) and the smallest with IGG-1 (*P* = 0.017). % area LNs with IGG-3 was also significantly higher than both IGG-1 (*P* < 0.0001) and IGG-2 (*P* = 0.001). In addition, IGG-2 was greater than IGG-1 (*P* = 0.013). Error bars show mean ± 95% CI

In order to further investigate whether the differences observed between vaccine-generated antibodies and NOV were due to differential detection of LBs and LNs, these features were quantified separately in the midbrain (SN) and temporal cortex (TC) of PD and DLB cases (Fig. 4). Since MSA cases do not have LBs and LNs, these cases were excluded from this analysis. Similarly, because clear and definable LBs are present only in the cortical grey matter and SN, the basal ganglia were not quantified for this analysis. The percentage area of LBs was determined based on their size and circularity. The remaining α-Syn immunoreactivity consisted mainly of LNs with some diffuse neuronal and neuropil staining.

There was no significant difference in levels of LB detection between the antibodies tested in the temporal cortex of PD cases (Fig. 4a). However, in the same brain region, the % area of LNs was highest with IGG-2 (0.15%, *P* < 0.0001) when compared to IGG-1 (0.08%), IGG-3 (0.03%) and NOV (0.01%). There was significantly more staining of LNs with IGG-1 than NOV (*P* < 0.0001), but no difference was observed between IGG-3 and NOV. Similarly, LN detection in the SN was significantly greater with IGG-1 (0.04%), IGG-2 (0.04%) and IGG-3 (0.05%) when compared to NOV (0.02%) with the highest staining achieved with IGG-3 (*P* < 0.0001).

In the TC of DLB cases (Fig. 4e), there were significantly more LBs detected by IGG-2 (0.06%, *P* = 0.001) and IGG-3 (0.06%, *P* < 0.006) than by NOV (0.02%). In addition, LB immunoreactivity with IGG-2 was higher than IGG-1 (0.04%, *P* = 0.014). Similarly, in the TC, immunoreactivity for LNs using IGG-2 (0.19%, *P* < 0.0001) was significantly greater than the immunoreactivity from IGG-1 (0.14%), IGG-3 (0.11%) or NOV (0.07%).

A similar relationship was observed in the SN of DLB cases (Fig. 4g, h) with IGG-2 (*P* = 0.001) and IGG-3 (*P* < 0.0001) demonstrating significantly higher LB and LN detection than IGG-1 and NOV. In this region, IGG-3 demonstrated a greater immunoreactivity for LBs than IGG-1 (*P* = 0.002) (Fig. 4g), as well as greater immunoreactivity for LNs than IGG-1 and IGG-2 (*P* < 0.001) (Fig. 4h).

### IGG-3 specifically recognises oligomeric and fibrillar forms of α-Syn in vitro

**Fig. 5:**
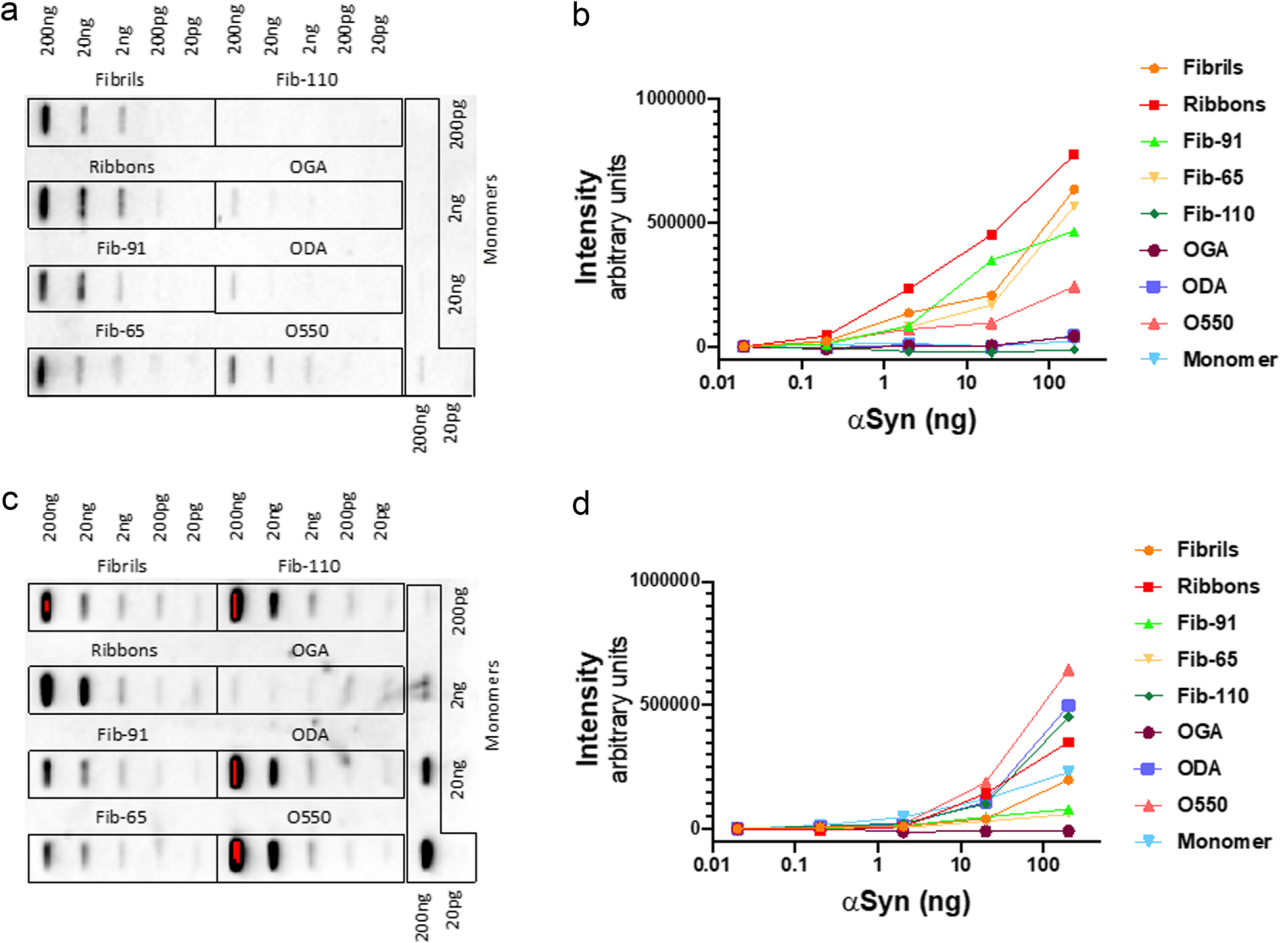
IGG-3 specifically binds to native oligomers and fibrillar a-Syn in vitro*.* **a** Slot blot showing the immunoreactivity of a-Syn strains to IGG-3. **b** Semi-quantitative analysis of the binding of IGG-3 to monomers, oligomers and fibrils. **c** Slot blot showing the immunoreactivity of a-Syn strains to Syn1. **d** Semi-quantitative analysis of the binding of Syn1 to monomers, oligomers and fibrils

We next assessed the affinity of IGG-3 antibodies, generated by vaccine VX-03, for structurally and functionally distinct, pure, oligomeric and fibrillar α-Syn assemblies immobilised on nitrocellulose membranes. The data presented in Fig. 5 show that IGG-3 binds to all fibrillar strains, with highest affinity for ribbons. It binds native oligomeric α-Syn with lower efficiency. No binding to glutaraldehyde, dopamine cross-linked oligomers nor to monomeric α-Syn is observed. Also, IGG-3 does not bind fibrils lacking the C-terminal 30 amino acid residues (Fib-110). In contrast, the commercial antibody Syn1 (clone 42, BD bioscience) binds to all α-Syn strains and to oligomers, except glutaraldehyde cross-links. It also binds to monomeric α-Syn. Its epitope is described to span over residues 91 to 96/99. Consistent with that, it binds fibrils lacking the C-terminal 30 amino acid residues (Fib-110).

## Discussion

This study demonstrated that purified IgG fractions collected from guinea pigs after immunisation with UBITh-based vaccines targeting α-Syn specifically recognised pathological forms of α-Syn in human brain tissue from PD, DLB and MSA cases. Moreover, our data indicate that the three vaccines tested in this study (VX-01, VX-02 and VX-03), which target different but overlapping epitopes in the α-Syn peptide, induced anti-α-Syn antibodies that demonstrate similar or greater immunoreactivity for α-Syn lesions than the commercial antibody NOV which is used widely for diagnostic purposes.

The vaccine-generated antibodies had a higher recognition of α-Syn aggregates compared to the NOV, suggesting that the amount of α-Syn pathology reported from neuropathological diagnosis may not be a comprehensive representation of the total pathology present. Further examination revealed that this was mainly due to additional recognition of LN pathology. The use of polyclonal antibodies compared to the monoclonal NOV antibody may contribute to the recognition of both oligomeric and fibrillar α-Syn, but also generally raises concerns for increased non-specific staining. However, the lack of positive staining in age-matched control brains further confirmed that the antibodies from each of the three vaccines were specific only to pathological α-Syn conformations found in LBs, LNs and GCIs/GNIs (Fig. 1).

### Amount of α-Syn recognition was epitope specific between diseases

#### IGG-3 preferentially recognises glial α-Syn in MSA

**Fig. 6:**
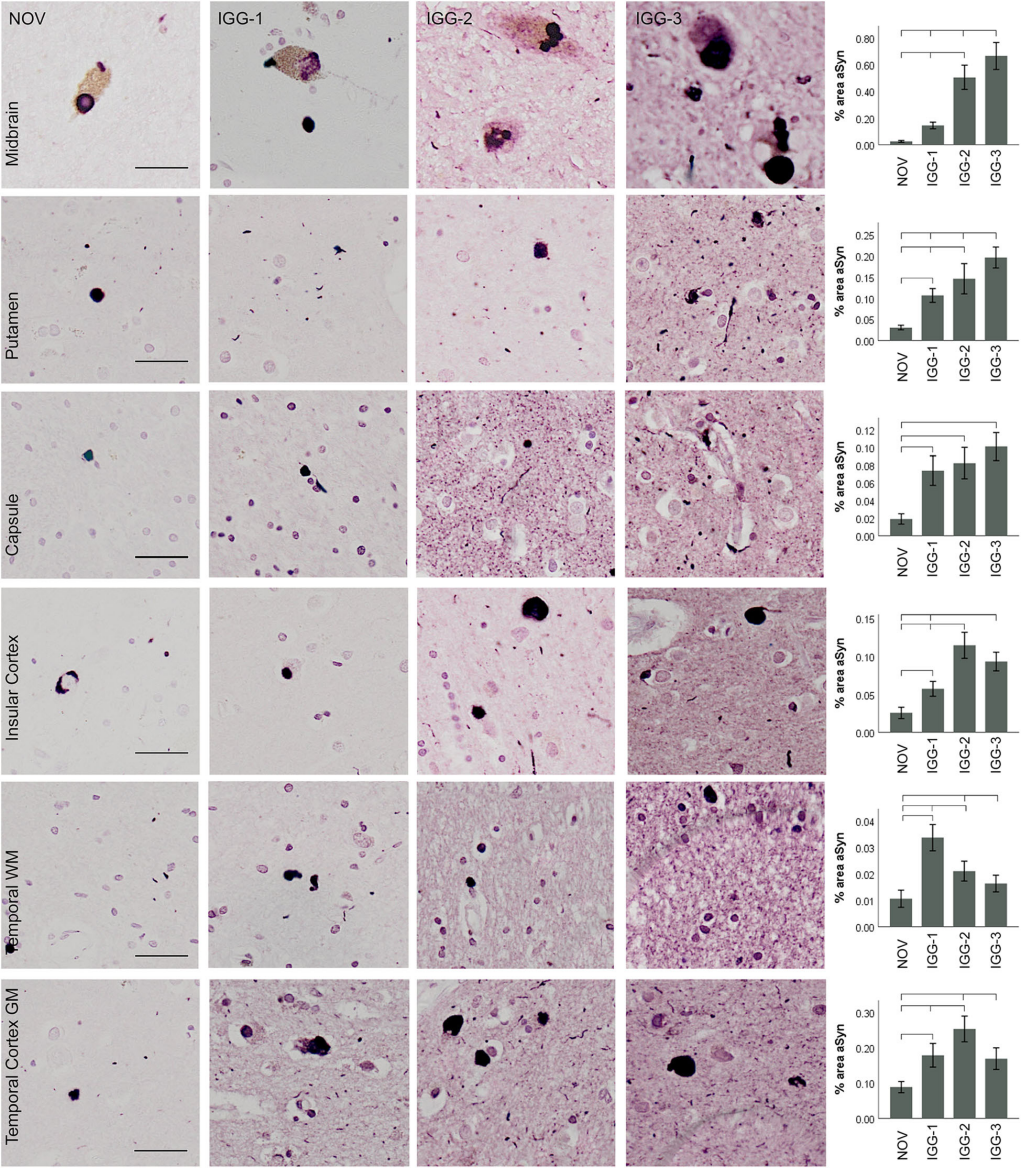
α-Syn immunoreactivity in dementia with Lewy bodies. Representative images and corresponding quantification of α-Syn immunoreactivity obtained using IGG-1, IGG-2, IGG-3 and NOV. α-Syn-positive inclusions were detected with both NOV and all 3 antibodies in each brain region. IGG-1, IGG-2 and IGG-3 detected a significantly higher quantity of α-Syn inclusions than NOV in all brain regions (except IGG-1 in substantia nigra) (*P* < 0.05). IGG-2 and IGG-3 detected a significantly higher quantity than IGG-1 in all brain regions except the cortical GM and internal capsule (*P* < 0.05). α-Syn detected by IGG-3 was significantly higher than IGG-2 in the substantia nigra, putamen and temporal cortex (*P* > 0.0001). Error bars show mean ± 95% CI. Scale bar = 100 μm

The synucleinopathies include the Lewy body diseases (PD and DLB) and MSA. LB diseases share the same hallmark of α-Syn aggregates (LBs and LNs), but in MSA the pathogenic α-Syn accumulates within glial cells rather than neurons. While the highest levels of α-Syn detection fluctuated between IGG-2 and IGG-3 in PD and DLB in different brain regions (Figs. 2 and 6), IGG-3 consistently recognised the highest levels of α-Syn pathology in cases of MSA (Fig. 3). This suggests that there may be a difference in the conformation of α-Syn aggregates between LB diseases and MSA, in accord with the different cell types affected. This was demonstrated by Trojanowski and Lee who found that the intracellular milieu of glial cells (GCIs) enables the formation of a more toxic strain of α-Syn when compared to neuronal intracellular environments [12]. GCIs were of a more ‘compact’ structure and were resistant to proteinase K digestion indicating that these aggregates had a different conformation to the typical LB-like structures [12].

In light of this, the present study found that IGG-3 preferentially recognises GCI strain over IGG-1 and IGG-2, whereas in PD and DLB cases, IGG-2 demonstrated higher levels of α-Syn detection in cortical brain regions. Further investigation would be required to assess whether the increased aSyn immunoreactivity from IGG-3 is due to increased number of glia or neurons with aSyn or just increased amount of aSyn within each cell.

### Amount of α-Syn recognition was epitope specific between brain regions in LB diseases

α-Syn pathology in LB diseases typically propagates from midbrain regions (including SN) towards the basal ganglia and then rostrally through the cortex [5]. In each region, α-Syn aggregates adopt different morphologies. In the SN, dopaminergic neurons develop characteristic eosinophilic LBs with a ‘halo-like’ appearance (demonstrated in Figs. 2 and 6 top panel). In contrast, cortical LBs appear as denser, less-spherical structures (Figs. 2 and 6). This suggests that the conformation of α-Syn aggregates or other LB components may be different in these brain regions [13].

In PD (Fig. 2) and DLB (Fig. 6), IGG-2 recognised the highest levels of α-Syn in cortical regions of the temporal lobe and insular cortex, but in other regions of the basal ganglia and in the SN, IGG-3 detected the most α-Syn pathology. IGG-2 did not detect higher levels of α-Syn than IGG-3 in the cortex of MSA cases (Fig. 3) suggesting that it preferentially recognises cortical neuronal LBs. According to Braak staging of LB diseases, the SN and basal ganglia are brain regions that are affected at a relatively early stage in the development of disease potentially putting the vaccine generating the IGG-3 at an advantage for targeting these earlier α-Syn inclusions and preventing their propagation to other brain regions.

In addition to LBs, α-Syn also aggregates within neurites in PD and DLB. While these diseases share a common underlying pathology, the α-Syn burden differs between brain regions with higher α-Syn loads being reported in limbic and neocortical regions in DLB compared to PD [4]. To assess whether the difference in α-Syn detection between IGG-1, IGG-2, IGG-3 and NOV was due to their ability to bind to LNs, the % area of α-Syn immunoreactivity was quantified for these structures separately in the SN and temporal cortex.

In the SN of PD cases, the most notable difference was between NOV and IGG-3 for LN detection with no difference in % area LBs (Fig. 4c, d). This suggests that the significantly higher level of α-Syn immunoreactivity with IGG-3 that was observed in this brain region was due to additional recognition of α-Syn epitopes in neuronal processes by IGG-3. In contrast, in the SN of DLB cases, both increased levels of LBs and LNs contribute to the higher levels of α-Syn immunoreactivity with IGG-3. The greatest difference was observed between IGG-3 and NOV for LN % area (0.32%) suggesting that the additional detection of LNs is the main contributor to the increased α-Syn immunoreactivity in the SN with IGG-3 (Fig. 6).

Differences in LB/LN detection may also explain the increased levels of α-Syn immunoreactivity with IGG-2 in the temporal cortex of PD and DLB cases (Figs. 2 and 6). In PD, there was no difference in LB detection between the antibodies; however, significantly higher levels of LNs were observed with IGG-2 compared to the other antibodies tested. While there was no trend in levels of LBs, the trend observed in % area LNs matched the total levels of α-Syn immunoreactivity in the temporal cortex, suggesting the effect of the antibodies is due to their ability to bind to LNs. Similarly, in the temporal cortex of DLB cases, the highest level of α-Syn immunoreactivity was observed with IGG-2 LN detection. However unlike in PD, IGG-2 and IGG-3 also detected significantly more LBs than NOV and IGG-1 suggesting that both LBs and LNs contribute to increased α-Syn immunoreactivity in this brain region. Variation in LB detection between antibodies directed against the C-terminus of α-Syn has also been previously reported in human brain tissue [34] and a recent study investigating the structure of α-Syn fibrils isolated from DLB and PD post mortem brains showed that the fibrils adopt different structures or “strains” in each disease type [35]. This may explain the different recognition of LBs/LNs with IGG-2 and IGG-3. In support of this, IGG-3 was found to bind with nanomolar affinity to pure fibrillar α-Syn polymorphs, and with much lower affinity to non-fibrillar pathway oligomeric species but not to monomeric α-Syn. It exhibited the highest affinity for the polymorph ribbons which was shown to trigger inclusions, characteristic of MSA, in oligodendrocytes upon injection into rodents [11]. These findings support the view that IGG-3 contains somewhat conformational antibodies, recognising best a structurally well-defined fibrillar α-Syn polymorph. Further investigation of conformational specific α-Syn antibodies by immunohistochemistry would provide additional insight to the conformational specificity of IGG-3; however, the conformational specificity of many currently used commercial antibodies has shown to be questionable in recent reports [36].

### Vaccine VX-03 as a potential candidate for immunotherapy

Immunotherapy in mouse models of LB disease has shown promising results over the years. Several studies using a passive immunisation approach have shown that antibodies are able to enter the brain [37, 38]; however, it is acknowledged that only a small percentage (~ 0.1%) of intravenous administered antibodies cross the blood-brain barrier [39]. This means that it is important to efficiently target the pathological forms of α-Syn in human brains. Once in the brain, anti-α-Syn antibodies have been shown to prevent α-Syn propagation [18, 34, 40] and ameliorate behavioural deficits [38] in mouse models with glial [24] or neuronal pathology [38]. Vaccines for synucleinopathies are in early phases of clinical development [9] with only two passive vaccines in phase II trials [30, 41]. Few of these studies have examined the target engagement of their antibodies in human tissue from LB disease cases. This study has demonstrated that IGG-3 (PD100806-09-I-2-Syn) purified from guinea pigs immunised with VX-03 effectively binds to pathological α-Syn aggregates in PD, DLB and MSA cases, particularly in brain regions affected at earlier stages of disease development. These findings support the selection of PD100806-09-I-2-Syn (UB-312) for phase I clinical trials. Further work in experimental models assessing the effect of this vaccine on α-Syn aggregation and functional consequences will provide additional information in support of the human trials.

### Limitations

The main limitation is that this is a study of the immune responses of the guinea pigs immune system directed against to the α-synuclein vaccines whereas the intended use is in humans. In addition, this study has provided in vitro evidence for the binding of vaccine-generated antibodies to sections of post mortem human tissue that has undergone prolonged formalin fixation, tissue sectioning and antigen retrieval protocols, whereas the effect of the vaccine needs to also be assessed in vivo.

## Conclusion

Retrospective analysis of the many AD clinical trials for immunotherapy highlights the importance for development of a vaccine targeting a denatured form of a self-protein/peptide with high immunogenicity while minimising the inflammatory response due to over activation of autologous T cells. The UBITh-platform allows the design of site-directed immunogens meeting these requirements in both the preclinical and clinical studies of United Neuroscience’s lead vaccine against amyloid-β, UB311 [28, 42]. The anti-α-Syn vaccines analysed in the present study demonstrated the generation of antibodies with high target engagement with pathological α-Syn in human tissue from various synucleinopathies, with high specificity for aSyn oligomers and fibrils but not monomers. IGG-3, generated by VX-03 vaccine (UB-312), was found to bind the highest level of α-Syn aggregates in brain regions that are affected relatively early in disease progression. This indicates that UB-312 is a promising candidate for treating synucleinopathies and is now undergoing phase 1 clinical trials for PD (NCT04075318).

## Supplementary information

**Additional file 1: Supplementary Figure S1.** limited background immunoreactivity to secondary antibodies by slot blot analysis. a) Omission of the primary antibody IGG-3 and using a Rabbit anti-Guinea pig IgG (H+L) secondary antibody-HRP demonstrated limited immunoreactivity. Faint staining was observed for fibrils and ribbons in a dose independent manner, indicating that the background signal most likely did not interfere with the clear dose response seen when applying IGG-3. b) Omission of the primary antibody Syn1 and using a Goat anti-Mouse IgG (H+L) secondary antibody-HRP conjugate demonstrated a clear lack of immunoreactivity.

## Data Availability

The datasets obtained and/or analysed during the current study available from the corresponding author on reasonable request.
