# Correction: Novel antibodies detect additional α-synuclein pathology in synucleinopathies: potential development for immunotherapy

**DOI:** 10.1186/s13195-022-01156-8

**Published:** 2023-01-21

**Authors:** Jacqui T. Nimmo, Ajay Verma, Jean-Cosme Dodart, Chang Yi Wang, Jimmy Savistchenko, Ronald Melki, Roxana O. Carare, James A. R. Nicoll

**Affiliations:** 1 Clinical Neurosciences. Clinical & Experimental Sciences, Faculty of Medicine, University of Southampton, Southampton, UK; 2 United Neuroscience, Dublin, Republic of Ireland; 3 Institute Francois Jacob (MIRCen), CEA and Laboratory of Neurodegenerative Diseases, CNRS, Paris, France

**Correction: Alz Res Ther 12, 159 (2020)**

10.1186/s13195-020-00727-x

Following publication of the original article [1], the authors reported a mistake in Fig. 4. Graph C must have been inadvertently copied into the place for graph A when making the figure. The corrected graph similarly did not show any significant relationships and does not change the interpretation of the results. The figure legend and description of the figure in the text remain correct.

**Fig. 4:**
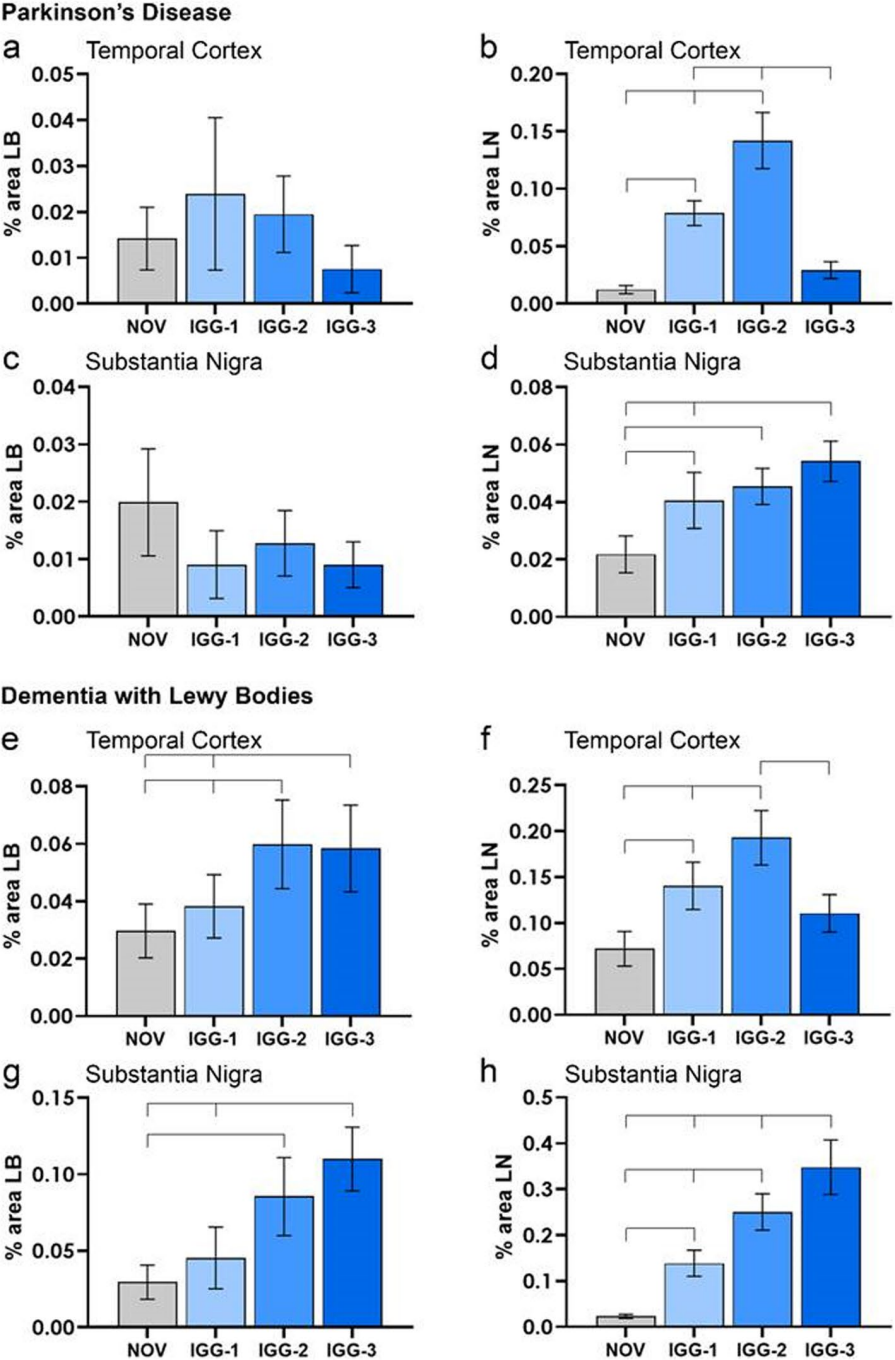
Quantification of Lewy bodies and Lewy neurites in PD and DLB. IGG-1, I GG-2, IGG-3. No difference in % area LBs in the temporal cortex and SN of PD cases was observed between each of the antibodies and NOV (**a**, **c**). **b** However, significantly more LNs were detected with IGG-2 and IGG-1 antibodies compared to IGG-3 and NOV (*P* < 0.0001); in the TC, there was no difference between IGG-3 and NOV. In addition, IGG-2 stained more LNs than IGG-1 in the temporal cortex of PD cases (*P* < 0.0001). **d** In the SN of PD cases, the % area LNs was greater for IGG-1 (*P* = 0.002), IGG-2 (*P* < 0.0001) and IGG-3 (*P* < 0.0001) compared to NOV. in addition IGG-3 was significantly greater than IGG-1 (*P* = 0.028) (**d**). **e** % area LBs detected by IGG-3 (*P* = 0.006) and IGG-2 (*P* = 0.001) was significantly greater than NOV. In addition, IGG-2 stained more LBs than IGG-1 in TC of DLB cases (*P* = 0.014). **f** Significantly more LNs were detected with IGG-2 compared to IGG-1, IGG-3 and NOV (*P* < 0.0001). In addition, IGG-1 stained more LNs than NOV in TC of DLB cases (*P* < 0.0001), but there was no difference between IGG-3 and NOV. **g** IGG-2 (*P* = 0.001) and IGG-3 (*P* < 0.0001) showed greater % area LBs in SN of DLB cases compared to NOV. IGG-3 also detected a higher level of LBs compared to IGG-1 (*P* = 0.002). **h** All antibodies showed significantly higher detection of LNs in SN compared to NOV in which the greatest difference occurred with IGG-3 (*P* < 0.0001) and the smallest with IGG-1 (*P* = 0.017). % area LNs with IGG-3 was also significantly higher than both IGG-1 (*P* < 0.0001) and IGG-2 (*P* = 0.001). In addition, IGG-2 was greater than IGG-1 (*P* = 0.013). Error bars show mean ± 95% CI

The original article [1] has been updated.

Below is the corrected Fig. 4.
