# Supplementary figures and images for: Novel antibodies detect additional α-synuclein pathology in synucleinopathies: potential development for immunotherapy
Source: Alzheimers Res Ther. 2020 Nov 30;12:159. doi: 10.1186/s13195-020-00727-x (PMC7702704; doi:10.1186/s13195-020-00727-x)

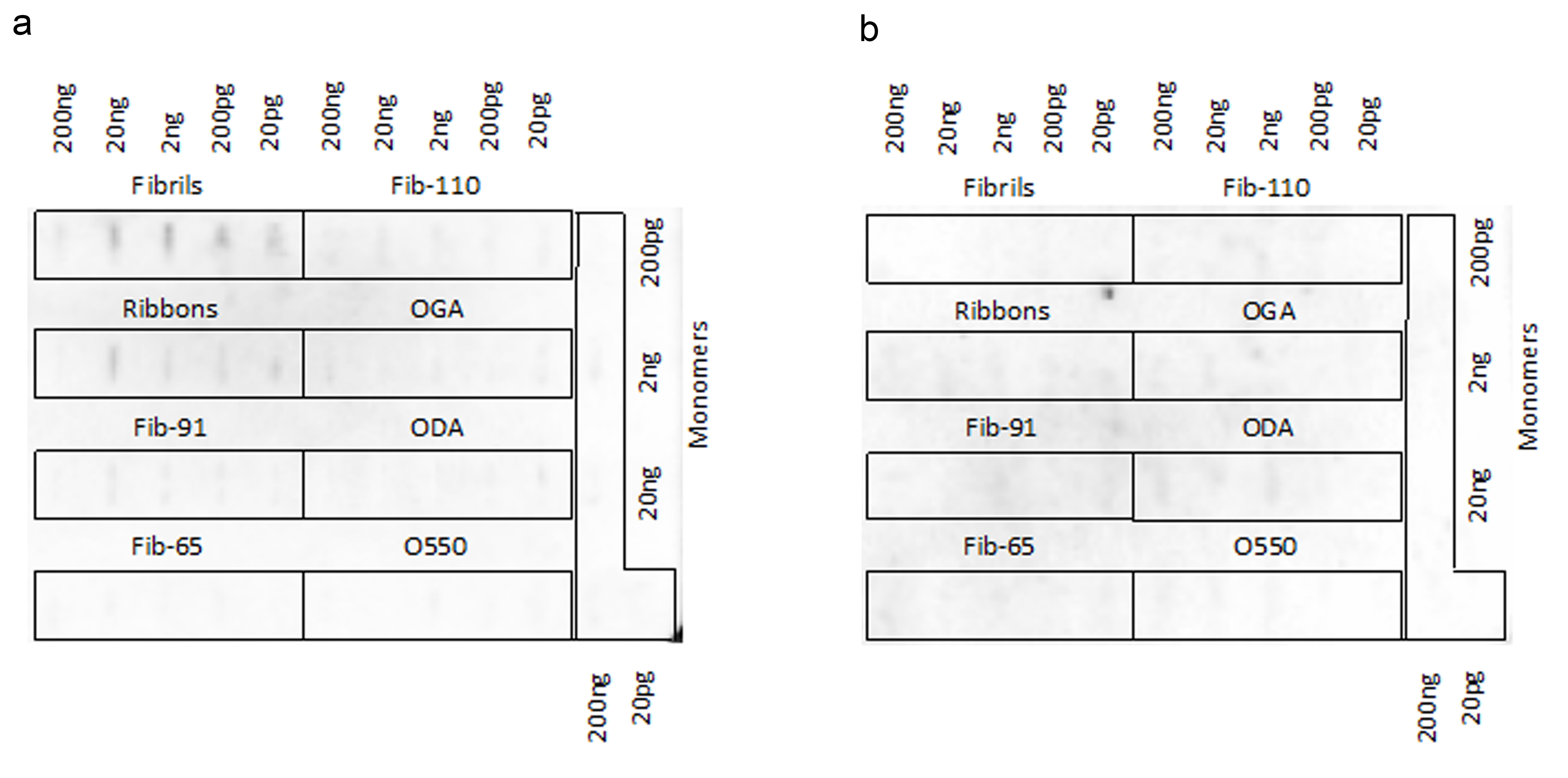

Supplement: Supplementary file 1 — Additional file 1: Supplementary Figure S1. limited background immunoreactivity to secondary antibodies by slot blot analysis. a) Omission of the primary antibody IGG-3 and using a Rabbit anti-Guinea pig IgG (H+L) secondary antibody-HRP demonstrated limited immunoreactivity. Faint staining was observed for fibrils and ribbons in a dose independent manner, indicating that the background signal most likely did not interfere with the clear dose response seen when applying IGG-3. b) Omission of the primary antibody Syn1 and using a Goat anti-Mouse IgG (H+L) secondary antibody-HRP conjugate demonstrated a clear lack of immunoreactivity. [file 13195_2020_727_MOESM1_ESM.tif]
